# Mind and Charcoal

**Published:** 1882-09

**Authors:** 


### MIND AND CHARCOAL.

The diamond, the most valuable thing in Nature, so sparkling, so beautiful and bright, whose lustre does not pale a particle in the lapse of ages, is but another condition of carbon, or charcoal, which you cannot touch without soiling your fingers; beautifully shadowing to us that greater change which shall come over the frail tenement of man, when it shall be raised a "a spiritual body," fit for the heavenly mansions, and destined to a beatific existence when time shall be no more. But the human mind cannot act without the agency of carbon, and by this same agency do the trees grow, and the flowers bloom, and the connection between these is called "The correlation of Mental and Physical Force," which phrase we were afraid to put at the head of this article, lest the reader should be frightened by its apparent obstruseness and skip it over; for all like the kind of reading best which requires the least thinking; the newspapers, civil, religious and mongrel, have found this out, and load their columns with all sorts of impossible fabrications, as weak as water and as wishy-washy as cold soup; but publishers know that "there is money in it," the thoughtless public are pleased, and down we are going, at railroad speed "ad infernum."

Carbon represents heat; vegetation grows by absorbing carbon; and the hotter the climate, the faster does vegetation grow. At the Poles there is no carbon, and there is no vegetation. When a tree is growing, it absorbs as much carbon as it will give out, when it is cut down and burned; if a pound of carbon, or wood, is burned and applied to water, so as to make steam, that steam, if economized, will raise a man to the top of Mount Washington. But if a man wants to go to the top of Mount Washington, he can raise himself up there by the force of his will, acting on his feet; but in order to do this, the brain must act upon the muscles of the body, and to do that, carbon must be supplied to it; this carbon is obtained from the food we eat; and unless we eat f[illegible]

which contains carbon, we will soon die, as the body gets cold; in a sense, freezes. Thus we see that carbon, acting on water, will raise a man sky-high; this is called "physical force;" carbon feeding the brain, enables a man to will himself to the same altitude, and away he goes, as fast as his legs will carry him; this is the result of "mental force;" and now the reader sees the connection between physical and mental force, that they accomplish the same result, and by the use of the same agency, heat, obtained from carbon, or charcoal. That is to say, the vital force of the body and of the vegetable, is generated by carbon. It would be useless to bother the reader with this long rigmarole, unless we could derive from it some practical lesson, by which we can be made better or happier. The largest specimens of vegetation and animals, grew in the earlier ages, in parts where the atmosphere was a furnace; and as the crust of the earth cools, both grow more slowly, and the time for dying comes before they reach as great a stature as of old; and so it must be with man, the more carbon he absorbs, the more food he can eat and appropriate healthfully to the bodily uses, the larger or stronger will he be, according to whether the greater amount of carbon is absorbed by the brain or muscles; it is the stomach which is to prepare the food for the elimination of the carbon contained in it; this process is called "digestion," hence, the more perfect, the more vigorous, the more healthful a man's digestion is, the more vigorous will he be in mind or body, if not both; so whatever we do to weaken, to disease the stomach, we do that much towards impairing mind and body; towards depraving the race; degrading it toward the mere animal and the idiot. If we eat just enough, both mind and body are invigorated; if we eat too little, both become weak and faint; the body trembles, the mind is inefficient; if we eat too much, the stomach cannot eliminate the material which is to give out a pure carbon, and it then gives out an impure article, and mind and body are oppressed; the former loses its activity, the latter its vigor. Farming, or any other active out-door life, tends to perfect digestion; city life, with its inactions and its intemperances impairs the digestion then follows the startling truth, and known to be truth, the world over, that families in cities, whole family names, die out in two or three generations; it has been stated that it rarely happens that a grand child reaches maturity in Paris; scarcely a dozen of the same prominent family names are found in the New York City Directories of 1878, which were in the directory of 1802, just two generations ago; and but for the replenishment of lads from the country, the progeny of hard out-door workers of vigorous stomachs, eliminating carbon largely, so as to give power to produce children of robust health, New York would be almost depopulated in a comparatively short time. These are serious truths, and to antagonize such results, let every child born in New York, and whose father and grandfather were born in New York, be sent to the country during the first month of its life, to be brought up to out-door labor, so as to renew the constitution. The intelligent reader will feel a very deep interest in these statements, and will regard them as general truths, to be modified by antagonizing circumstances, but not the less true and practical for all that. Let us recapitulate. As much heat or carbon is absorbed by a tree during its growth, as it will give out when it is burned; so, as much bodily and nervous energy will be given out by a man, as the carbon contained in the food which he eats will supply.

But it does not follow that the more a man eats, the more carbon will he absord, and consequently, the larger, stronger, and more intellectual will he become; these depend on the healthful vigor of his digestion, because it is this which prepares the food for the separation of the carbon in it, previous to its absorption into the system; and as an active out-door life is the best means known for securing a perfectly healthful digestion, the inference is fair, logical and legitimate, and observation will prove its truthfulness, that out-door activities, for the first thirty years of life, at least, are very certain to be followed by high health, bodily power, intellectual ability and long life; this intellectual activity being greater or less, according to the greater or less size of the brain proper, which is that portion which lies in the front and upper region of the head.

The mind acts on the body through the brain, making the brain in the nature of a machine, whose working involves waste, and the necessity of repair or renewal, as oil to the wheels of vehicles of locomotion; this renewal is made from the food we eat; the faster a physical machine runs, the faster will it wear out, and there is no help for it; but the human machine had Divinity for its architect, and it does not follow that the faster or more vigorously it works, the more intense the thoughts and sensations, the sooner will it decay; but it only follows that the harder a man works, or thinks, or the more intense are his sensations, the more nourishment must be given to the muscles which work, and to the brain, through which comes our sensations; that is, the more carbon must be supplied to the system; and as was before noticed, that the greater the amount of carbon supplied, the larger was the tree, the greater the animal, the more vigorous the action of the brain the mental work, it therefore follows that the human machine increases its physical and mental capabilities by the very increase of its activities; that the more a man works, the more and better he can work; the more he thinks, the more and better he can think; hence, the busiest men live the longest, whether it be physical or mental industry; thus, Newton, and others of the greatest intellects in physics, in theology, and in ethics, have lived to a good old age.

But it is a beautiful thought, and suggestive, too, that man expends his carbon in two directions; through the muscles, enabling him to work a great deal; and through the brain, enabling him to think a great deal; if expended equally in these two directions, a man becomes a good worker and a good thinker; but if he would become the best worker, the excess of carbon must be expended through the muscles; if, on the other hand, he desires to excel in the world of thought, he must expend the greater share of his carbon through the brain.

But another beautiful thought must not be omitted. A good digestion takes the carbon out of the food eaten and throws it into the circulation, the blood; but throwing coal into a furnace will not warm the house, the fire must be kindled; the coal must burn, and its burning gives out heat, this is called combustion ; the body is the furnace, the carbon put into it by eating, is its coal or fuel, but it must be kindled, must be set on fire, by having oxygen introduced ; we know that a fire will not burn unless the air can get to it and supply it with its oxygen ; so, also, will not the carbon in the blood kindle into warmth and heat, unless plenty of good air is introduced into it, which is done by breathing it into the lungs, where all the blood goes, and so, being brought into contact there, the oxgen of the air and the carbon of the blood join, and combustion is the result, giving out heat, fire, warmth, and as the out-door air is the purest, freshest and best ; the more we are out of doors, the more oxygen we get, the more perfectly the carbon is burned, and the greater the amount of healthful heat is there in the system.

We all know that the harder we work, the sooner we get tired and the more hungry we become ; and students at school, and academy, and college, know very well that they grow weak by hard study ; and that their appetites become so imperative and exacting sometimes, late at night, that remorseless contributions have been made on neighbor's corn-cribs, dairies, orchards, melon-patches, and henneries. Who does not now feel that we have made "the correlation of the mental with the physical forces" as plain as a pike-staff, and very interesting, too ; that shows our genius. Reader, don't you feel that it is a plain matter, after all ? Any body can make an egg stand on end, after a Columbus has shown him once how to do it ! ! But O, how little of the immeasureable world of truth does any man know, do all men know ! Balloons for ordinary traveling purposes, may yet be contrived ; some may think that a man may, sometime, travel as fast as a telegram, and who knows but that the science of "mind and charcoal" may be so systematized, that a man may prepare himself for a specified amount of labor, by eating a specific food of a specific quantity, may graduate the intensity of his sensations by the measure of his meat.

